# Data of *in vitro* synthesized dsRNAs on growth and development of *Helicoverpa armigera*

**DOI:** 10.1016/j.dib.2016.04.026

**Published:** 2016-04-16

**Authors:** Yojana R. Chikate, Vishal V. Dawkar, Ranjit S. Barbole, Priyadarshini V. Tilak, Vidya S. Gupta, Ashok P. Giri

**Affiliations:** Plant Molecular Biology Unit, Division of Biochemical Sciences, CSIR-National Chemical Laboratory, Dr. Homi Bhabha Road, Pune 411008, Maharashtra, India

**Keywords:** RNAi, dsRNA, *H. armigera*, Gene silencing, Pest control

## Abstract

The data presented in this article is related to the research article “RNAi of selected candidate genes interrupts growth and development of *Helicoverpa armigera*” (Chikate et al., 2016) [1]. RNA interference (RNAi) is emerging as a potent insect pest control strategy over current methods and their resistance by pest. In this study we tested 15 different *in vitro* synthesized dsRNAs for gene silencing in *Helicoverpa armigera*. These dsRNAs were specific against *H. armigera* enzymes/proteins such as proteases like trypsins (*HaTry2*, *3, 4* and *6*), chymotrypsin (*HaChy4*) and cysteine proteases such as cathepsin (*HaCATHL*); glutathione S-transferases (*HaGST1a, 6* and *8*); esterases (*HaAce4, HaJHE*); catalase (*HaCAT*); super-oxide-dismutase (*HaCu/ZnSOD*); fatty acid binding protein (*HaFabp*) and chitin deacetylase (*HaCda5b*). These dsRNAs were fed to second instar larvae at an optimized dose (60 µg/day) for 3 days separately. Effects of dsRNA feeding were observed in terms of larval mass gain, percentage mortality and phenotypic abnormalities in later developmental stages of *H. armigera*. These findings might provide potential new candidates for designing sequence-specific dsRNA as pesticide in crop protection.

**Specialization Table**

**Table:** 

Subject area	Biology
More specific subject area	RNAi and Biochemistry
Type of data	Tables and Figures
How data was acquired	Workflow of primer designing*, in vitro* dsRNA synthesis and its stability
Data format	Raw
Experimental factors	We selected major enzyme/protein classes in *H. armigera* for gene silencing by feeding them specific dsRNA
Experimental features	Each dsRNA fed to 30 insects
Data source location	CSIR-National Chemical Laboratory, Pune, India
Data accessibility	Data is presented in this article

**Value of the data**

• This data will be useful to the other investigators to follow the same system for implementing in the field experiments by using nanoparticles for the stability of dsRNA.
• Environmental parameters need to be integrated in these experiments hence environmental factors can be explored for its concrete use in the ground level.
• The data support the development of further experiments on the use dsRNA in other devastating insect pests.
• Findings from this work will be valuable for designing potent dsRNA as pesticide in crop protection.

## 1 Data

Here, we demonstrated the use of dsRNA in pest control and various aspects *viz*. dsRNA stability, concentration and amplicon size were assessed based on ingestion by *Helicoverpa armigera* (Figs. 1 and 2). In the data, 15 dsRNAs targeting various *H. armigera* enzymes/proteins have been shown in terms of larval mass reduction post 96 and 144 h of dsRNA exposure (DPE) (Chikate et al. [1]). These dsRNAs were seen to be played role at larval and pupal level by reduction in mass (Fig. 3A, B and C). Upon ingestion of dsRNA by *H. armigera*, reduction in larval mass, abnormal phenotypes, egg laying capacity, gene silencing and enzyme activity pattern was successively studied.

## 2 Experimental design, materials and methods

### 2.1 Sequence analysis and primer designing

Based on earlier studies in our laboratory [2], [3], [4] we selected major enzyme/protein classes in *H. armigera* for gene silencing by feeding them specific dsRNA. These involved 15 mRNAs belonging to proteases (6), glutathione *S*-transferases (3), esterases (2), catalase (1)/super-oxide-dismutase (SOD) (1), fatty acid binding protein (1) and chitin deacetylase (1). Green fluorescent protein (GFP) was used as a negative control while glyceraldehydes phosphate dehydrogenase from *H. armigera* (GAPDH) was used as positive control. Sequence analyses for each of these target mRNA class was performed using CLUSTAL W and primers containing gene-specific sequence adapted to T7 promoter sequence in inverted orientation were designed manually (Fig. 1). These were around 45 bp long primers, devoid of self-complementarily or secondary structures giving amplicon of around 200–500 bp. The primer sequences, amplicon sizes for preparation of dsRNA of each mRNA are listed in Table 1. To check the effect of gene silencing at transcript level qRT-PCR analysis of cognate mRNAs was performed using primers designed in between amplicon region used for dsRNA synthesis. The primer sequences, amplicon sizes for qRT-PCR analysis of each mRNA are listed in Table 2.

### 2.2 *in vitro* dsRNA synthesis

cDNA synthesized from mRNA of AD and pigeon pea fed larvae and was used for amplification of target regions. These were cloned into pGEMT, confirmed and then linearlized plasmids were used as template for *in vitro* synthesis of dsRNA (Fig. 2A and B). The protocol used for *in vitro* dsRNA synthesis was based on MegaScript RNAi kit instruction manual (Ambion, Austin, Texas, USA). Once synthesized, dsRNAs were purified and assessed quality and quantity by agarose gel electrophoresis and Nanodrop 1000 spectrophotometer (Thermo scientific, Waltham, MA, USA).

### 2.3 Artificial diet preparation

Artificial diet (AD) was prepared by the method of Nagarkatti and Prakash [5]. List of ingredients which require for preparing artificial diet (AD) is mentioned in Table 3.

#### 2.3.1 Stability study of dsRNA

Prior to dsRNA feeding experiments, stability of dsRNA was assessed for different time intervals by coating dsRNA on AD cube. dsRNA targeting *HaGAPDH* and *HaTry*3 were used for this analyses. dsRNA (60 µg) was coated on surface of AD cube (~1 cm^2^) and allowed to diffuse dsRNA in the diet. These cubes were kept at control conditions for insect growth (24 °C temperature and 65% humidity). RNA was extracted from these cubes by using Trizol reagent (Invitrogen, Carlsbad, CA, USA) after 6, 12, 24 and 36 h of dsRNA coating. The quality, quantity and stability of RNA were determined by agarose gel electrophoresis and spectrophotometric analysis using Nanodrop (Thermo Scientific). It was found that dsRNA was stable up to 24 h (Fig. 2C). These dsRNAs were then used for feeding bioassays of *H. armigera*.

### 2.4 Insect culture

*H. armigera* larvae were procured from Division of Insect Ecology, ICAR-National Bureau of Agricultural Insect Resources, Bangalore, India. Larvae were maintained under laboratory conditions at humidity 65%, temperature 26 °C and photoperiod of 16 h light: 8 h dark. For homogeneity, insects were maintained in the lab for one generation and then used for experiments.

### 2.5 dsRNA feeding to *H. armigera*

In order to affirm the effect of chosen dsRNAs’ on growth and development of *H. armigera* upon feeding, bioassays were carried out as described below. We examined delivery of dsRNA through AD to find out better method, dsRNA solution was applied onto surface of AD cube (~1 cm^2^). dsRNA was allowed to diffuse into diet and fed to insects. In this way, dsRNA (60 µg/cube of AD) feeding was carried out for selected 17 genes and AD coated with DEPC treated water was used as non-dsRNA control. dsRNA targeting GFP was used as negative control and one targeting GAPDH as a positive control. dsRNA applications were done thrice at 24 h intervals for all target genes, fresh AD cube with fresh 60 µg application was used each time. Following to this, larvae were reared on normal AD and observed for their growth and development till they became pupa. During dsRNA feeding experiments, 25 larvae from each of the treatment groups were harvested in liquid nitrogen at 2, 4 and 8 days post first dsRNA exposure (DPE). Silencing effects were observed in terms of larval mass gain, percent mortality, phenotypic abnormalities, egg laying and reduction of target transcript expression as well as enzyme activity studies. Larval growth was found to be significantly retarded in most of the candidate dsRNA fed insects. This was evident from the reduction in mass gain of the dsRNA fed larvae unlike the control group (Fig. 3A and B). This was also evident from the distinct reduction in weight of pupae (Fig. 3C) developed upon three doses of the dsRNAs with interval of 24 h.

### 2.6 Data collection

To see the growth performance of *H. armigera* upon feeding of dsRNA*,* larvae were weighed after 24 h of first dose of dsRNA till pupation.

### 2.7 Statistical analysis

Data was analyzed by one-way analysis of variance (ANOVA) and *post-hoc* analyses using a Tukey–Kramer multiple comparisons test. Standard deviation and then standard error means were used to compare the replicates.

## Appendix A Supplementary material

Supplementary material

Supplementary material supplementary **Fig. 1.** Workflow for primer designing for dsRNA synthesis for *H. armigera* target genes. The basis of primer design for *HaGST* (six isoforms) is depicted in the figure. **Fig. 2.** Gel image for target regions of selected *H. armigera* candidate genes. The amplified product was purified and run on 2% agarose gel electrophoresis, specific bands corresponding to expected amplicons of target genes were obtained (**A**). *in vitro* dsRNA synthesis for selected target regions (**B**); synthesis was done using MegaScript RNAi kit, specific size of dsRNA was obtained. Stability of dsRNA upon coating on cube of AD at different time intervals; dsRNA targeting *HaGAPDH* and *HaTry*3 were used for this study (**C**). **Fig. 3.** Larval mass gain (gm) of *H. armigera* on DAE of dsRNA. Reduction of mass gain was significant for *HaAce4*, *HaJHE*, *HaCAT*, *HaCuZnSOD* and *HaCda5b* dsRNA fed larvae on 96 h DAE (**A**) and 144 h. (**B**), respectively. Pupal mass of dsRNA fed *H. armigera* larvae on 10 DAE to dsRNA (**C**).

## Footnotes

Appendix A Supplementary data associated with this article can be found in the online version at doi:10.1016/j.dib.2016.04.026.
