# Supplementary figures and images for: Data of in vitro synthesized dsRNAs on growth and development of Helicoverpa armigera
Source: Data Brief. 2016 Apr 16;7:1602–5. doi: 10.1016/j.dib.2016.04.026 (PMC4865661; doi:10.1016/j.dib.2016.04.026)

## Slide 1
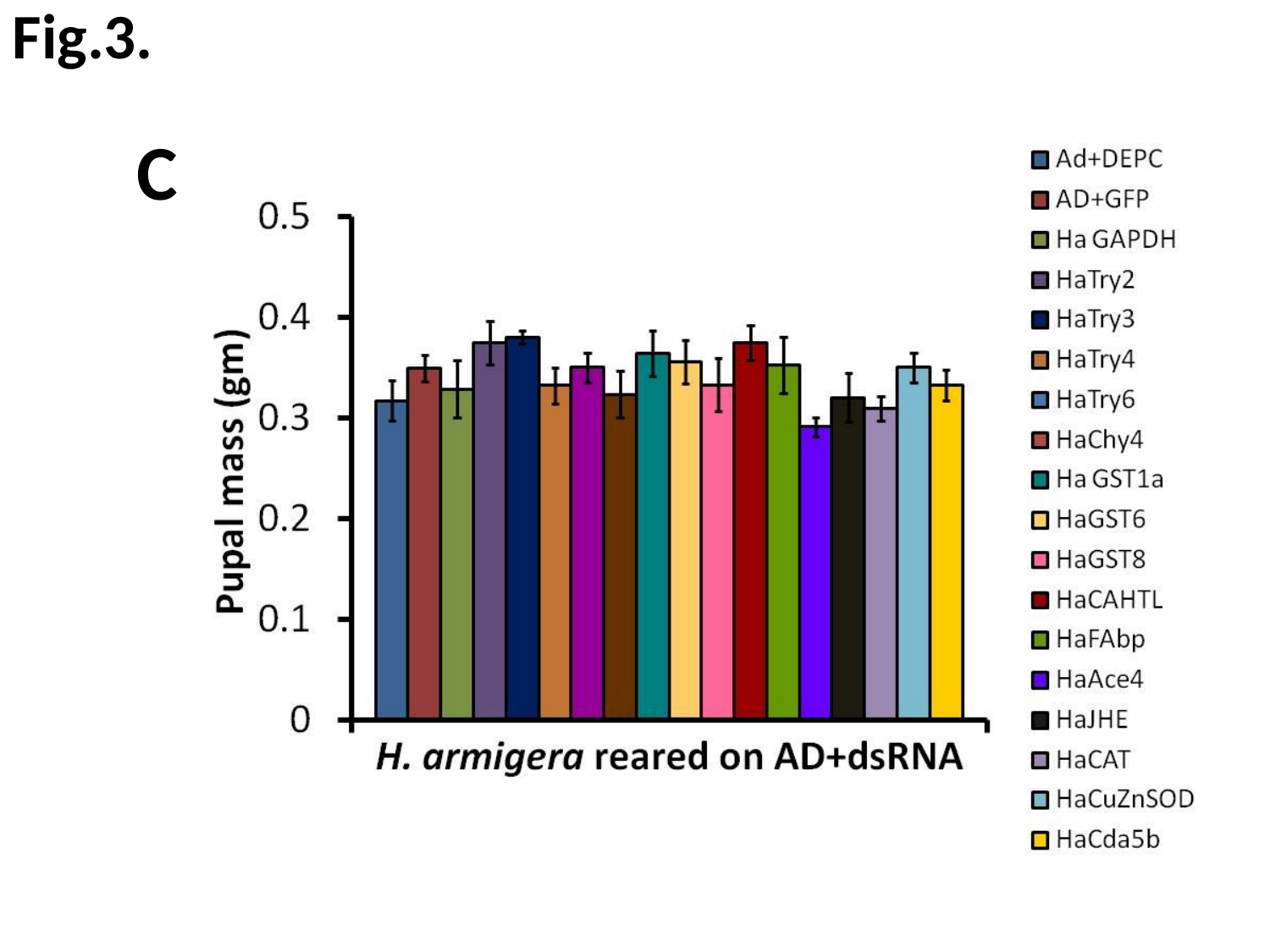

Fig.3.
C

Supplement: Supplementary file 2 — Supplementary material supplementary Fig. 1. Workflow for primer designing for dsRNA synthesis for H. armigera target genes. The basis of primer design for HaGST (six isoforms) is depicted in the figure. Fig. 2. Gel image for target regions of selected H. armigera candidate genes. The amplified product was purified and run on 2% agarose gel electrophoresis, specific bands corresponding to expected amplicons of target genes were obtained (A). in vitro dsRNA synthesis for selected target regions (B); synthesis was done using MegaScript RNAi kit, specific size of dsRNA was obtained. Stability of dsRNA upon coating on cube of AD at different time intervals; dsRNA targeting HaGAPDH and HaTry3 were used for this study (C). Fig. 3. Larval mass gain (gm) of H. armigera on DAE of dsRNA. Reduction of mass gain was significant for HaAce4, HaJHE, HaCAT, HaCuZnSOD and HaCda5b dsRNA fed larvae on 96 h DAE (A) and 144 h. (B), respectively. Pupal mass of dsRNA fed H. armigera larvae on 10 DAE to dsRNA (C). [file mmc2.zip › Supplementary Figures/Supplementary Figure 3C.pptx]

## Slide 1
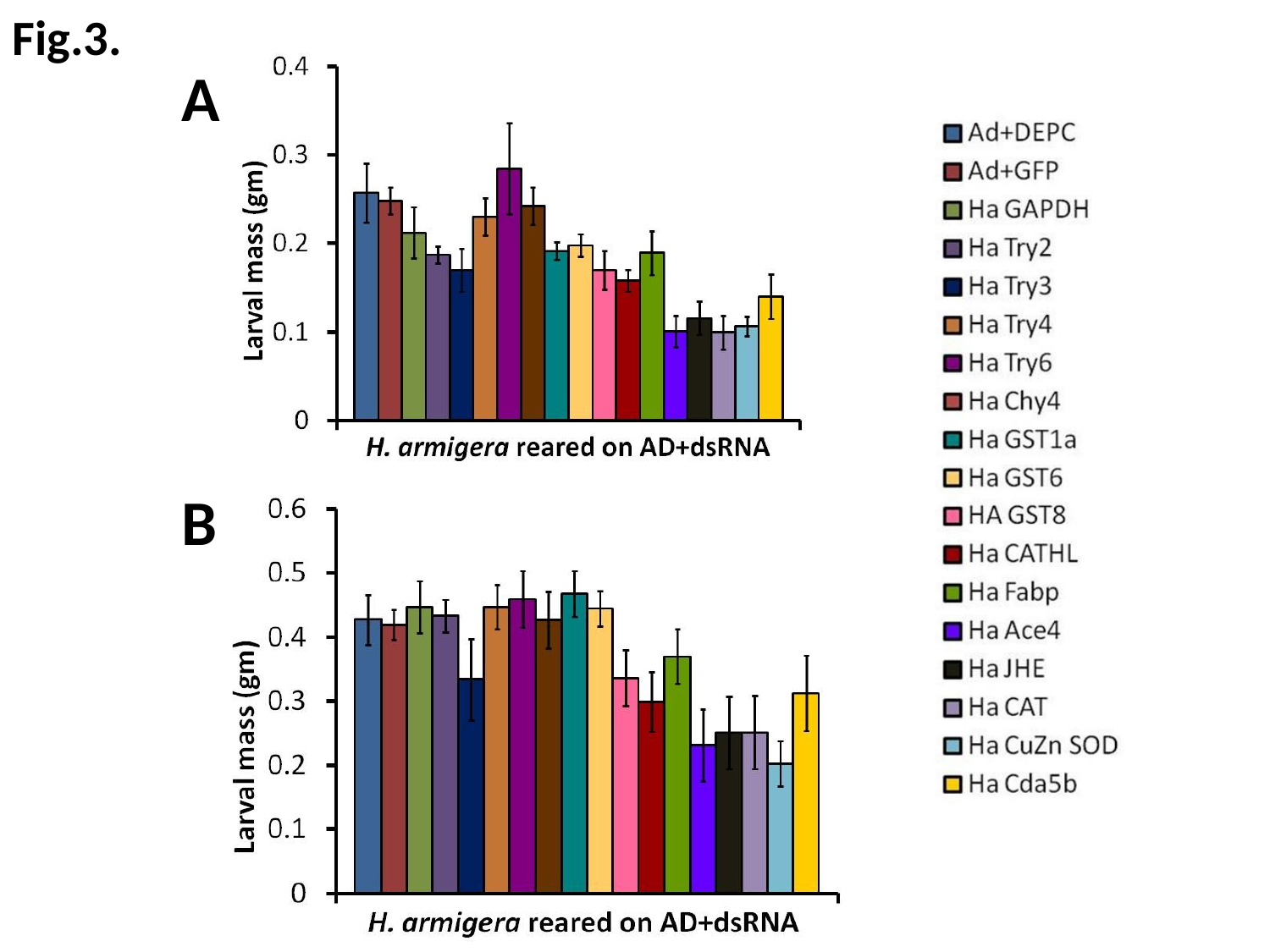

Fig.3.
A
B

Supplement: Supplementary file 2 — Supplementary material supplementary Fig. 1. Workflow for primer designing for dsRNA synthesis for H. armigera target genes. The basis of primer design for HaGST (six isoforms) is depicted in the figure. Fig. 2. Gel image for target regions of selected H. armigera candidate genes. The amplified product was purified and run on 2% agarose gel electrophoresis, specific bands corresponding to expected amplicons of target genes were obtained (A). in vitro dsRNA synthesis for selected target regions (B); synthesis was done using MegaScript RNAi kit, specific size of dsRNA was obtained. Stability of dsRNA upon coating on cube of AD at different time intervals; dsRNA targeting HaGAPDH and HaTry3 were used for this study (C). Fig. 3. Larval mass gain (gm) of H. armigera on DAE of dsRNA. Reduction of mass gain was significant for HaAce4, HaJHE, HaCAT, HaCuZnSOD and HaCda5b dsRNA fed larvae on 96 h DAE (A) and 144 h. (B), respectively. Pupal mass of dsRNA fed H. armigera larvae on 10 DAE to dsRNA (C). [file mmc2.zip › Supplementary Figures/Supplementary Figures 3 AB.pptx]
