# Supplementary material for: Data of in vitro synthesized dsRNAs on growth and development of Helicoverpa armigera
Source: Data Brief. 2016 Apr 16;7:1602–5. doi: 10.1016/j.dib.2016.04.026 (PMC4865661; doi:10.1016/j.dib.2016.04.026)
Supplement: Supplementary file 3 — Supplementary material [file mmc3.zip › Supplementary Tables/Supplementary Table 1.docx]

**Table 1.** List of primers used for *in vitro* dsRNA synthesis for selected *H. armigera* genes

| Sr. No | Primer Name | Primer sequence (5'→3') | F/R | Accession No. |
| --- | --- | --- | --- | --- |
| 1 | HaAce4_iF | TAATACGACTCACTATAGGGAGAGTGGAGACTCAACGAAGATC | F | JF894118 |
| 2 | HaAce4_iR | TAATACGACTCACTATAGGGAGACTCTTAGACCACATAATGAACTC | R |  |
| 3 | HaCAT_iF | TAA TAC GAC TCA CTA TAG GGA GAGACCATGGCTCAAGCAGAGAGC | F | JQ009332 |
| 4 | HaCAT_iR | TAA TAC GAC TCA CTA TAG GGA GATTGTGGTCCATCACGTTGGTAG | R |  |
| 5 | HaCathL_iF | TAA TAC GAC TCA CTA TAG GGA GATATGACGCTAGGCTCGAAGGTC | F | EU528473 |
| 6 | HaCathL_iR | TAA TAC GAC TCA CTA TAG GGA GACTAGGGCAACCTTCAAAGCTCC | R |  |
| 7 | HaCda5b_iF2 | TAA TAC GAC TCA CTA TAG GGA GA TCTGCACTCCATCAGCCATAAG | F | GQ411191 |
| 8 | HaCda5b_iR2 | TAA TAC GAC TCA CTA TAG GGA GA ATCATGCAATCTTGCTCCGAAG | R |  |
| 9 | HaChy4_iF | TAATACGACTCACTATAGGGAGAGCTGGTCTTGTGATCACCATC | F | Y12273 |
| 10 | HaChy4_iR | TAATACGACTCACTATAGGGAGAGATGGTCACAGAGCTCAACTG | R |  |
| 11 | HaCu/ZnSOD_iF | TAA TAC GAC TCA CTA TAG GGA GATCTGCGGGTGCTCACTTCAAC | F | JQ009331 |
| 12 | HaCu/ZnSOD_iR | TAA TAC GAC TCA CTA TAG GGA GA GATAACACCGCAGGCAATACG | R |  |
| 13 | HaFabp_iF | TAA TAC GAC TCA CTA TAG GGA GA TCACCTCCTCCACCTTCAAGAC | F | EU325560 |
| 14 | HaFabp_iR | TAA TAC GAC TCA CTA TAG GGA GA ACATCCTTGGCGGTCATCACAG | R |  |
| 15 | HaGAPDH1_iF | TAATACGACTCACTATAGGGAGACAAGGCTGGTGCTGAATACG | F | JF417983 |
| 16 | HaGAPDH1_iR | TAATACGACTCACTATAGGGAGACAGAGGGTCCATCCACTG | R |  |
| 17 | HaGFP1i_F | TAATACGACTCACTATAGGGAGA CAAGATACCCAGATCATATGAAAC | F | L29345 |
| 18 | HaGFP1i_R | TAATACGACTCACTATAGGGAGA GCTTCCATCTTTAATGTTGTGTC | R |  |
| 19 | HaGST1a_iF **^#^** | TAATACGACTCACTATAGGGAGAGGAGACAATATCCAAGAGG | F | HM209431 |
| 20 | HaGST1a_iR | TAATACGACTCACTATAGGGAGACGTTGATCCTCTGCAAGAG | R |  |
| 21 | HaGST6_iF | TAATACGACTCACTATAGGGAGAGACAAACTGACCTTGGCAGAC | F | GQ149104 |
| 22 | HaGST6_iR | TAATACGACTCACTATAGGGAGAACTGCGCTACCATAGCTCTG | R |  |
| 23 | HaGST8_iF | TAATACGACTCACTATAGGGAGAGAGAACAGCACAAAGAAG | F | FJ546089 |
| 24 | HaGST8_iR | TAATACGACTCACTATAGGGAGAGCCAAGAACTTCTCAGCG | R |  |
| 25 | HaJHE_iF | TAA TAC GAC TCA CTA TAG GGA GA CCACCAAGATCTACACGGACC | F | HM588760 |
| 26 | HaJHE_iR | TAA TAC GAC TCA CTA TAG GGA GA ATTCCTGTTCCGCTCATCAAG | R |  |
| 27 | HaTry2_iF | TAATACGACTCACTATAGGGAGAATGGAGAGACCAAATTCGGAG | F | EU770391 |
| 28 | HaTry2_iR | TAATACGACTCACTATAGGGAGACCAGTAGCGAAGCACCTG | R |  |
| 29 | HaTry3_iF | TAATACGACTCACTATAGGGAGAGTTCAGTGGTGGCTCGCTG | F | EU325548 |
| 30 | HaTry3_iR | TAATACGACTCACTATAGGGAGAGCGTGGAATGTGCAGACCTC | R |  |
| 31 | HaTry4_iF | TAATACGACTCACTATAGGGAGACGACATCAACTACCGTCGTG | F | EF600059 |
| 32 | HaTry4_iR | TAATACGACTCACTATAGGGAGACAGAGGACCACCAGAGTC | R |  |
| 33 | HaTry6_iF | TAATACGACTCACTATAGGGAGAGCAGTCCACAACGTTGCTTCG | F | Y12276 |
| 34 | HaTry6_iR | TAATACGACTCACTATAGGGAGACGATGCCGTTGTGGTAGAGAG | R |  |

**#** This primer pair was designed targeting six GST isoforms HM209429, HM209427, HM209428, HM209430, EF591059 and one mentioned in above table
