# Supplementary material for: Data of in vitro synthesized dsRNAs on growth and development of Helicoverpa armigera
Source: Data Brief. 2016 Apr 16;7:1602–5. doi: 10.1016/j.dib.2016.04.026 (PMC4865661; doi:10.1016/j.dib.2016.04.026)
Supplement: Supplementary file 3 — Supplementary material [file mmc3.zip › Supplementary Tables/Supplementary Table 2.docx]

**Table 2.** List of primers used for real time PCR analyses of selected target genes

| Sr. No. | Primer Name | Primer sequence- 5'-3' | F/R | Accession No. |
| --- | --- | --- | --- | --- |
| 1 | HaAce4 F | ATTGTCCCCTCTGTCAAGGAAC | F | JF894118 |
| 2 | HaAce4 R | GATATAATAGCCCAAGGTGCGG | R |  |
| 3 | HaCAT F | CCCCGAGGATTTGCTGTTAAA | F | JQ009332 |
| 4 | HaCAT R | GATGAAGAAAATTGGCGTGTTGT | R |  |
| 5 | HaCATHL F | CTGGGCCACTTACAACGAAGAT | F | EU528473 |
| 6 | HaCATHL R | GATCCCAACCAGGAGTTGAACT | R |  |
| 7 | HaCda5b F | GCCTTCATCAGATTCCTCAACC | F | GQ411191 |
| 8 | HaCda5b R | CACCCAGTCAATCACTTCGCT | R |  |
| 9 | Hachy 4F | TGACTTGTCAGGTGGCCAAGCTG | F | GU323796.1 |
| 10 | Hachy 4R | GCGATTCTGGTACCGCCGGAGAAC | R |  |
| 11 | HaCu/ZnSOD F | CTGACCCTGATGACCTTGGAG | F | JQ009331 |
| 12 | HaCu/ZnSOD R | GATAACACCGCAGGCAATACG | R |  |
| 13 | HaFabp F | CTTGAAGCAGGTGCAGAAGTCA | F | EU325560 |
| 14 | HaFabp R | ACAGCCTTCATCTCTTCGGGT | R |  |
| 15 | HaGAPDH 1F | TGCTGAATACGTCGTTGAATCC | F | JF417983 |
| 16 | HaGAPDH 1R | TTCTTAGCACCACCCTCTAAATGAG | R |  |
| 17 | HaGFP F | CACTGGAGTTGTCCCAATTCTTG | R | L29345 |
| 18 | HaGFP R | CCTTCACCCTCTCCACTGACAG | F |  |
| 19 | HaGST 1F **^#^** | CAAAATGAAGGGTCTGGGAGAA | F | HM209431.1 |
| 20 | HaGST 1R | CGTTCAAAGCGTATGTCTTCGA | R |  |
| 21 | HaGST 6F | CTCGTGGCGACTGTGTCCACTA | F | GQ149104.1 |
| 22 | HaGST 6R | AGTCGCTTTCACCAGCTCAAACC | R |  |
| 23 | HaGST 8F | TCTTTACCCAGCTGATCCGAAA | F | FJ546089.1 |
| 24 | HaGST 8R | AAAGCTGGAAACAGAATCCCAC | R |  |
| 25 | HaJHE F | GCAAAAACGTGCTGAGACTGG | F | HM588760 |
| 26 | HaJHE R | TCCTTGATGATGCTGCTCTGAC | R |  |
| 27 | HaTry 2F | GCGTAAAGGATGCGGTTGG | F | EU770391 |
| 28 | HaTry 2R | CAGGATGGCAACCATCCATG | R |  |
| 29 | HaTry 3F | CGACCACACTGACGCGAG | F | EU325548 |
| 30 | HaTry 3R | GCACGCCACTGGACATGG | R |  |
| 31 | HaTry 4F | GTGCTACCCCTTCTGATTC | F | EF600059 |
| 32 | HaTry 4R | AACTTGTCGATGGAGGTGAC | R |  |
| 33 | HaTry 6F | TGGCTGGGGTGACACTTTCT | F | Y12277 |
| 34 | HaTry 6R | GTCTCCCTGGCACTGGTC | R |  |

**#** This primer pair was designed for six GST isoforms HM209429, HM209427, HM209428, HM209430, EF591059 and one mentioned in above table
