# Supplementary material for: Data of in vitro synthesized dsRNAs on growth and development of Helicoverpa armigera
Source: Data Brief. 2016 Apr 16;7:1602–5. doi: 10.1016/j.dib.2016.04.026 (PMC4865661; doi:10.1016/j.dib.2016.04.026)
Supplement: Supplementary file 3 — Supplementary material [file mmc3.zip › Supplementary Tables/Supplementary Table 3.docx]

**Table 3.** List of ingredients to prepare artificial diet (AD)

| Component A | |
| --- | --- |
| Bengal germ | 12.5 gm |
| Yeast extract | 3.00 gm |
| Wheat germ | 1.25 gm |
| Casein | 0.875 gm |
| Sorbic acid | 0.125 gm |
| Methylparaben | 0.25 gm |
| Water | 37.50 ml |
| Component B |  |
| Choline chloride | 0.0875 gm |
| Streptomycin sulphate | 0.005 gm |
| Ascorbic acid | 0.50 gm |
| Cholesterol | 0.0375 gm |
| Multivitamin capsule | 0.25 |
| Vitamin E capsule | 0.125 |
| Water | 7.50 ml |
| Component C | |
| Agar | 1.625 gm |
| Water | 45.00 ml |
